# Accuracy Improvement of Binocular Vision Measurement System for Slope Deformation Monitoring

**DOI:** 10.3390/s20071994

**Published:** 2020-04-02

**Authors:** Qijun Hu, Ziyuan Feng, Leping He, Zihe Shou, Junsen Zeng, Jie Tan, Yu Bai, Qijie Cai, Yucheng Gu

**Affiliations:** 1 School of Civil Engineering and Geomatics, Southwest Petroleum University, Chengdu 610500, China; huqijunswpu@163.com (Q.H.); 201822000546@stu.swpu.edu.cn (Z.F.); tanjiepaper@126.com (J.T.); by_duo@163.com (Y.B.); guyucheng0728@163.com (Y.G.); 2 Sichuan Tibetan Area Expressway Co., Ltd., Chengdu 610041, China; ziheshou@163.com; 3 School of Mechatronic Engineering, Southwest Petroleum University, Chengdu 610500, China; 201911000062@stu.swpu.edu.cn; 4 School of Transportation and Logistics, Southwest Jiaotong University, Chengdu 610031, China; caiqijieswjt@my.swjtu.edu.cn

**Keywords:** binocular vision, accuracy analysis, slope deformation monitoring, structure parameters, environment parameters

## Abstract

This paper studies the limitations of binocular vision technology in monitoring accuracy. The factors affecting the surface displacement monitoring of the slope are analyzed mainly from system structure parameters and environment parameters. Based on the error analysis theory, the functional relationship between the structure parameters and the monitoring error is studied. The error distribution curve is obtained through laboratory testing and sensitivity analysis, and parameter selection criteria are proposed. Corresponding image optimization methods are designed according to the error distribution curve of the environment parameters, and a large number of tests proved that the methods effectively improved the measurement accuracy of slope deformation monitoring. Finally, the reliability and accuracy of the proposed system and method are verified by displacement measurement of a slope on site.

## 1. Introduction

Landslides are among the most common and catastrophic natural hazards, causing numerous casualties and fatalities every year [1]. Extracting the slope deforming information can effectively predict and warn of landslides early [2]. Historically, various monitoring methods have been employed to measure the slope deformation, including geodesy [3], GPS [4], INSAR [5], 3D laser scanning [6] and digital photogrammetry [7,8]. However, the construction industry seeks a more intelligent but low-cost technology to achieve dynamic and high-accuracy monitoring. Binocular vision technology is a promising new approach to deal with slope deformation monitoring [9] which has been widely and increasingly used in bridge vibration [10], multipoint displacement measurement [11], materials strain detection [12], depth estimation [13], and target tracking [14].

The accuracy of binocular vision technology is limited by the camera calibration parameters [15], system structure parameters [16], and environment parameters [17,18]. In order to eliminate the influence of camera calibration parameters on the monitoring, Li et al. [19] proposed a high-precision camera calibration method based on a multiview template and alternative bundle adjustment. Liu et al. [20] devoted to minimize the error between the geometric relation of 3D reconstructed feature points and the ground truth. Jin et al. [21] improved the calibration accuracy of the camera through distortion correction. Yang et al. [22] studied the influence of camera distortion, number of calibration images, number of targets and position of calibration board on the calibration accuracy. For system structure parameters, it mainly includes the angle between the camera optical axis and the baseline, baseline distance and monitoring distance [22,23,24]. Lu et al. [25] built the relationship between structure parameters (baseline, focal length and object distance) and distance measurement errors. Deng et al. [26] have studied the effect of vision measurement parameters on the accuracy of binocular reconstruction at high temperatures. Xu et al. [27] determined the optimal configuration of the system by comprehensively analyzing the baseline distance and measurement distance. Tang et al. [28] analyzed the relationship between the depth errors, the field of views and the distances through measurement tests, and obtained the error correction curves. Xu et al. [29] deduced the best relationship that the virtual baseline distance is 1–1.5 times of measurement distance for the double reflection system.

The camera calibration and system structure parameters are artificially adjustable. In contrast, the influence of environment parameters only can be optimized by image processing technologies based on monitoring data. The most popular image sharpening technology can be divided into two categories: image enhancement technology based on image processing and image restoration technology based on a physical model. Image enhancement technology [30] directly processes the pixel points of the image without considering the reasons of image quality deterioration so as to improve the vision effect of the image, such as histogram equalization [31], curvelet transform [32], and homomorphic filtering algorithm [33]. Image restoration technology [34] employs some prior knowledge of the degradation phenomenon to realize the process of establishing and inversely deducing degraded images so as to restore the original appearance of images, such as a physical model of atmospheric scattering [35], guided filtering [36] and improved mean filtering algorithm [37]. However, there is very little research into the above parameters of the binocular vision technology for slope deformation monitoring.

The main purpose of this study aims to investigate structure parameters and environment parameters for improving the accuracy of the binocular vision measurement system for slope deformation monitoring. The error distribution curves were obtained through laboratory tests. The sensitivity of structure parameters was analyzed, and parameter selection criteria were proposed. In addition, the environmental influences were optimized by image processing technology. Furthermore, field tests were conducted to verify the reliability and accuracy of the proposed system and method.

## 2. Binocular Vision Measurement System

### 2.1. Laser Rangefinder-Camera Station

**Figure 1:**
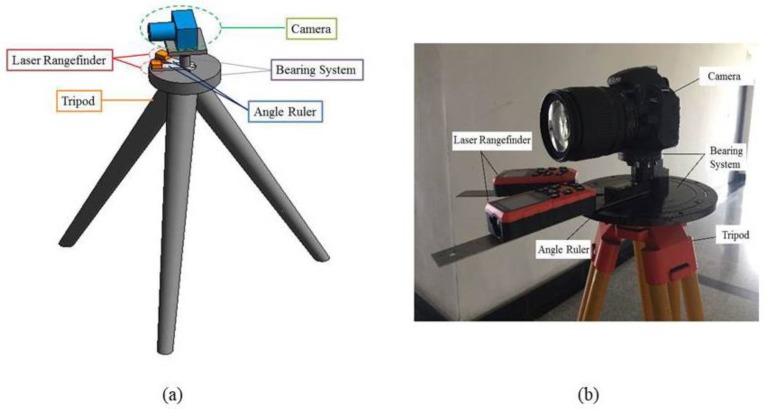
Binocular vision measurement system: (**a**) image of 3D simulation; (**b**) site layout diagram.

Figure 1 shows the binocular vision measurement system (BVMS) for slope deformation monitoring. In order to complete the site layout of the BVMS accurately and quickly in engineering monitoring, we design a simple laser rangefinder-camera station. It consists of tripod, laser rangefinders, camera, and angle measuring ruler. Laser rangefinders and the angle-measuring ruler are installed between the camera and the tripod. The laser rangefinder is fixed on the angle-measuring ruler, which is convenient for measuring the baseline distance and monitoring distance. One end of angle measuring ruler can be hinged according to the specific field of view. In order to make the layout system universal, the base of the design can be connected to the tripod through screws and the lower tray fixed the angle measuring ruler and the laser rangefinders. The camera can be connected to the tray using screws, and the camera direction can be flexibly rotated according to requirements. The connection is equipped with horizontal bubbles, which can be leveled during monitoring.

### 2.2. Slope Deformation Monitoring Based on BVMS

**Figure 2:**
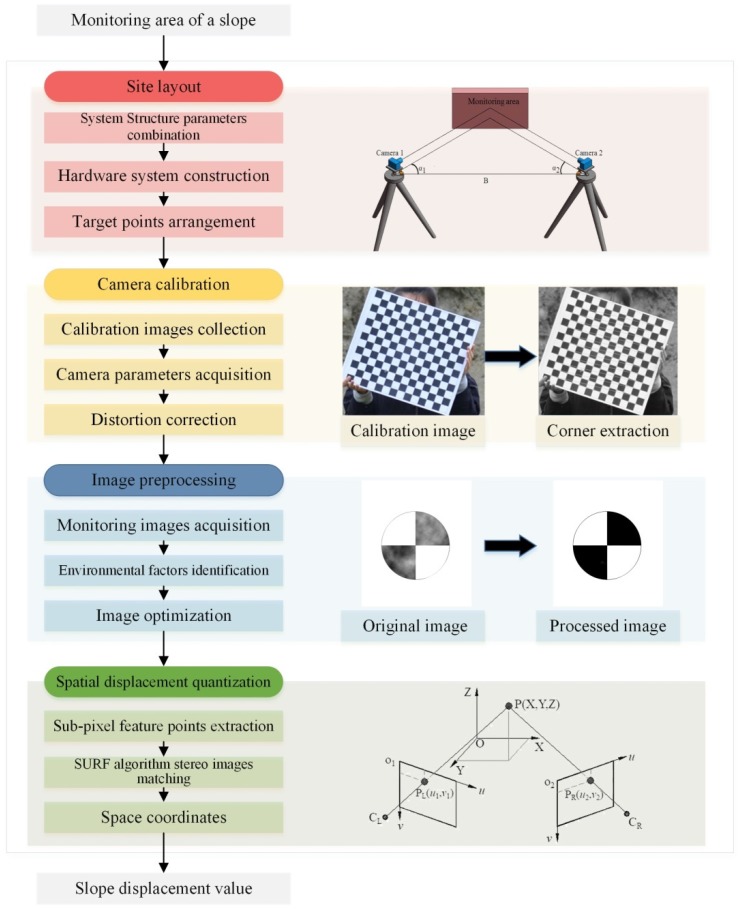
Slope deformation monitoring process based on binocular vision measurement system.

Figure 2 illustrates the process of slope deformation monitoring based on BVMS. Specific steps are as follows:

Step 1: Site layout. The laser rangefinder-camera station hardware system is built according to the system structure parameters, and then the target points are reasonably arranged in the monitoring area based on the field of view. 

Step 2: Camera calibration. The improved Zhang’s calibration method [38] is used to obtain camera parameters and achieve distortion correction of the camera. It should be noted that during the subsequent tests, refocusing the camera has been avoided. 

Step 3: Image preprocessing. The frequency of image capture can be set according to monitoring requirements, such as acquiring images every half an hour. Multiple (≥5) images should be taken continuously within one second for each acquisition, which can effectively reduce random errors during subsequent processing of the data. Then the environmental factors in the images are identified through human intervention. Finally, the preprocessing system designed in this paper is used to optimize the images. 

Step 4: Spatial displacement quantization. Feature points are extracted from the optimized images [39]. Three-dimensional information of the target points is converted by stereo matching with the Speeded-Up Robust Features (SURF) algorithm [40]. Then the displacement is calculated.

## 3. Accuracy Analysis of System Structure Parameters

### 3.1. System Monitoring Model

**Figure 3:**
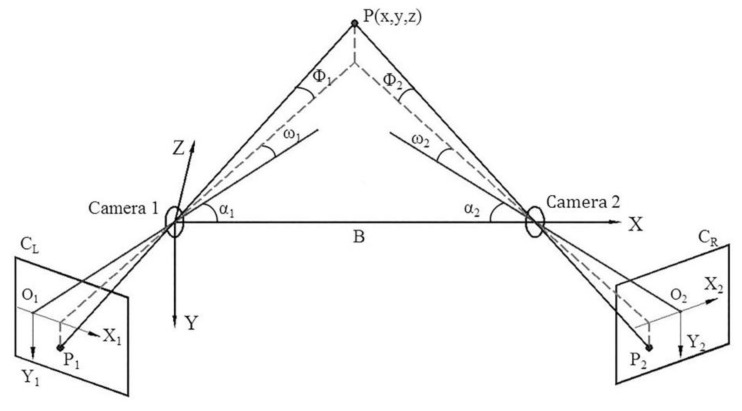
The monitoring model of binocular vision measurement.

Figure 3 presents the space geometry model of the BVMS [41]. $C_{L}$ and $C_{R}$ are the imaging planes of the left and right cameras. The image coordinate system of the left camera is $X_{1}O_{1}Y_{1}$, and the right one is $X_{2}O_{2}Y_{2}$. $P_{1}\left(X_{1},Y_{1}\right)$ and $P_{2}\left(X_{2},Y_{2}\right)$ are the image coordinates of target point P. $\alpha_{1}$ and $\alpha_{2}$ are the angles between the baseline and the optical axis of the left and right camera, respectively. The focal length is f. The projection geometry of the target point P on the left and right cameras is described by the horizontal field of view angle $\omega_{1}$ and $\omega_{2}$, and the vertical field of view angle $\Phi_{1}$ and $\Phi_{2}$. If the two cameras are placed horizontally and the coordinate system of the left camera is considered as the reference coordinate system, the coordinates of target point P(x,y,z) in the reference coordinate system can be calculated by Equation (1):

(1) $$x=\frac{B\cot(\omega_{1}+\alpha_{1})}{\cot(\omega_{1}+\alpha_{1})+\cot(\omega_{2}+\alpha_{2})},\;y=Y_{1}\frac{Z\sin\omega_{1}}{f_{1}\sin(\omega_{1}+\alpha_{1})}=Y_{2}\frac{Z\sin\omega_{2}}{f_{2}\sin(\omega_{2}+\alpha_{2})},\;z=\frac{B}{\cot(\omega_{1}+\alpha_{1})+\cot(\omega_{2}+\alpha_{2})},$$

where $\tan\Phi_{1}=\frac{Y_{1}}{f_{1}}\cos\omega_{1}$, $\tan\Phi_{2}=\frac{Y_{2}}{f_{2}}\cos\omega_{2}$, $\tan\omega_{1}=\frac{X_{1}}{f_{1}}$, $\tan\omega_{2}=\frac{X_{2}}{f_{2}}$.

Therefore, the position information of the target point P in the world coordinate system depends on the system structure parameters, including the monitoring distance Z, the baseline distance B, the angle α between the optical axis and the baseline, and the focal length f of camera. Equation (1) can be expressed into the vector function relationship, as shown in Equation (2):

(2) $$P\left(x,y,z\right)=F\left(B,\alpha ,f,X_{1},Y_{1},X_{2},Y_{2},Z\right),$$

According to the error analysis theory [42], the position information error of monitoring target point P in the world coordinate system:

(3) $$\Delta x=\sum_{k}\frac{\partial F_{x}}{\partial k}\delta_{k};\;\Delta y=\sum_{k}\frac{\partial F_{y}}{\partial k}\delta_{k};\;\Delta z=\sum_{k}\frac{\partial F_{z}}{\partial k}\delta_{k},$$

where $\delta_{k}$ is the error generated by each influencing parameter, k represents one of parameters with $B,\alpha ,f,X_{1},Y_{1},X_{2},Y_{2},Z$. By synthesizing the errors Δx, Δy and Δz in three directions, the comprehensive monitoring error Δ of position information can be obtained as:

(4) $$\Delta =\sqrt{{\left(\Delta x\right)}^{2}+{\left(\Delta y\right)}^{2}+{\left(\Delta z\right)}^{2}}=\sqrt{{\sum_{i}\sum_{k}\left(\frac{\partial F_{i}}{\partial k}\delta_{k}\right)}^{2}},$$

where i stands for the direction of x, y, z. The error transfer function is introduced to rewrite Equation (4) as:

(5) $$\Delta =\sqrt{{\sum {}_{k}\left(\psi_{k}\delta_{k}\right)}^{2}},$$

where $\psi_{k}$ represents the error transfer function of each parameter and is defined as:

(6) $$\psi_{k}=\sqrt{{\sum_{i}\left(\frac{\partial F_{i}}{\partial k}\right)}^{2}},$$

According to Equations (1)–(6), the error transfer function of space coordinates of the target point P can be shown as Equations (7)–(10):

(7) $$\{\begin{array}{l} \frac{\partial x}{\partial X_{1}}=-\frac{Z^{2}\cot\left(\omega_{2}+\alpha_{2}\right)\cos^{2}\omega_{1}}{Bf_{1}\sin^{2}\left(\omega_{1}+\alpha_{1}\right)}\\ \frac{\partial x}{\partial X_{2}}=-\frac{Z^{2}\cot\left(\omega_{1}+\alpha_{1}\right)\cos^{2}\omega_{2}}{Bf_{2}\sin^{2}\left(\omega_{2}+\alpha_{2}\right)} \end{array},$$

(8) $$\{\begin{array}{l} \frac{\partial z}{\partial X_{1}}=\frac{Z^{2}\cos^{2}\omega_{1}}{Bf_{1}\sin^{2}\left(\omega_{1}+\alpha_{1}\right)}\\ \frac{\partial z}{\partial X_{2}}=\frac{Z^{2}\cos^{2}\omega_{2}}{Bf_{2}\sin^{2}\left(\omega_{2}+\alpha_{2}\right)} \end{array},$$

(9) $$\{\begin{array}{l} \frac{\partial y}{\partial X_{1}}=\frac{Y_{1}Z\cos^{2}\omega_{1}}{Bf_{1}\sin^{2}\left(\omega_{1}+\alpha_{1}\right)}\\ \frac{\partial y}{\partial X_{2}}=\frac{Y_{2}Z\cos^{2}\omega_{2}}{Bf_{2}\sin^{2}\left(\omega_{2}+\alpha_{2}\right)} \end{array},$$

(10) $$\{\begin{array}{l} \frac{\partial y}{\partial Y_{1}}=\frac{Z\sin\omega_{1}}{f_{1}\sin\left(\omega_{1}+\alpha_{1}\right)}\\ \frac{\partial y}{\partial Y_{2}}=\frac{Z\sin\omega_{2}}{f_{2}\sin\left(\omega_{2}+\alpha_{2}\right)} \end{array},$$

### 3.2. Model Verification

A simulation test of slope slide was designed to verify the feasibility of the proposed system and further to analyze the influence of the structure parameters described above on the binocular system monitoring.

**Figure 4:**
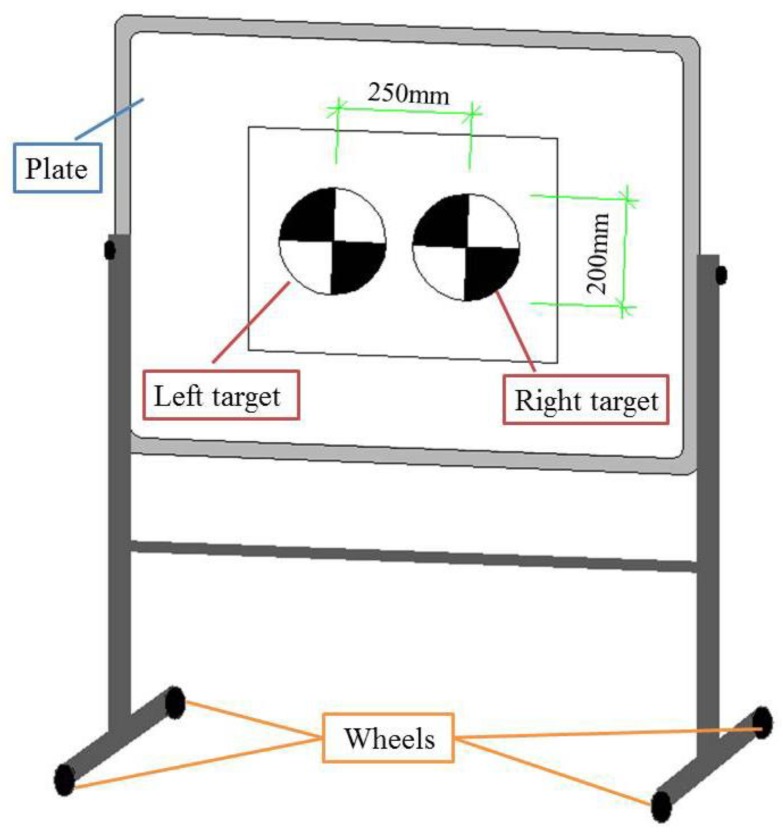
Diagram of removable platform of sliding deformation simulation test.

We used the laser rangefinder-camera station mentioned above, where the camera is a Nikon D3100 digital camera with resolution of 4608 × 3072 and 14.2 million pixels. The homemade calibration board is a chessboard with 14 × 13 blocks, where each block is 30 mm × 30 mm. Considering that displacement errors may be caused by manually moving it, we printed two identical targets with a diameter of 200 mm on A2 paper to simulate displacement deformation. Their center distance is 250 mm, that is, the true value of the displacement is 250 mm. To facilitate movement, we pasted the targets on the removable platform, as shown in Figure 4. During the tests, the camera had to be recalibrated for every set of structure parameters changed. At each position, five images of the targets were collected in succession to calculate the distance between the two targets.

**Figure 5:**
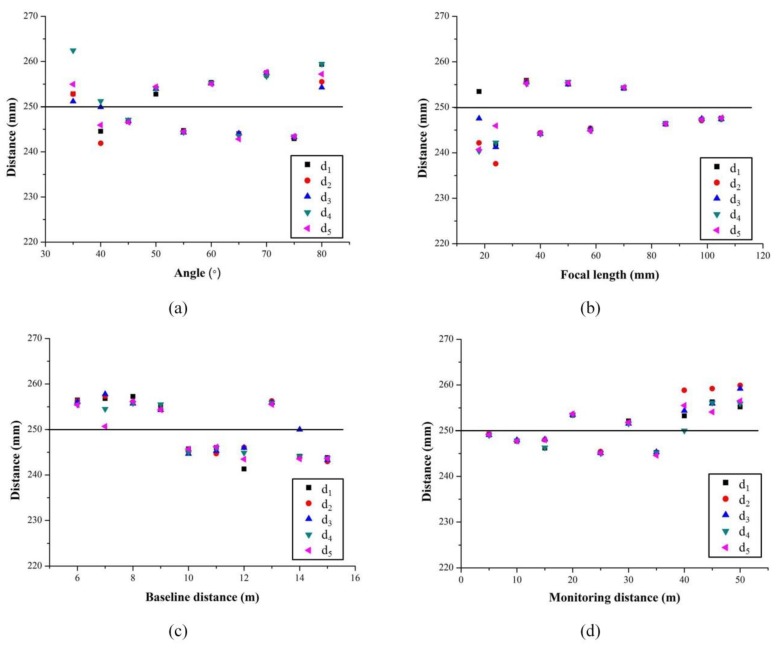
The total displacements measured by binocular vision measurement system with different structure parameters: (**a**) the angle α between optical axis and baseline; (**b**) focal length f; (**c**) baseline distance B; (**d**) monitoring distance Z.

The central location was performed on the targets in each image to obtain their pixel coordinates. Then the camera parameters were calibrated to convert the three-dimensional space coordinates. The center distance of the two targets was calculated according to Equation (11). Under each set of structure parameter combinations, there are five measured displacement values, as shown in Figure 5, which are $d_{1}$, $d_{2}$, $d_{3}$, $d_{4}$ and $d_{5}$.

(11) $$d_{i}=\sqrt{{\left(x_{li}-x_{ri}\right)}^{2}+{\left(y_{li}-y_{ri}\right)}^{2}+{\left(z_{li}-z_{ri}\right)}^{2}},$$

where $d_{i}$ is the measured displacement value; $\left(x_{l},y_{l},z_{l}\right)$ and $\left(x_{r},y_{r},z_{r}\right)$ are the three-dimensional coordinates of the center of left and right targets in the same image, respectively.

**Figure 6:**
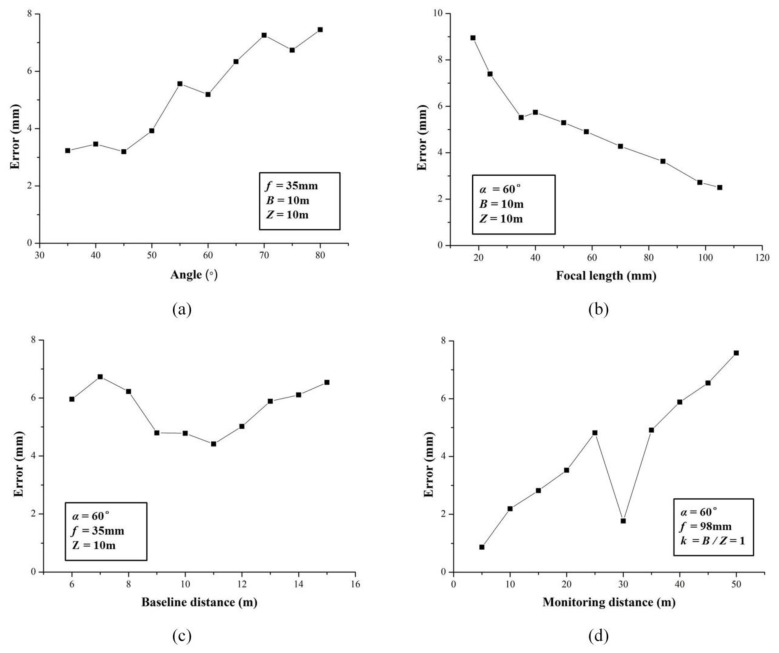
Distribution curve of displacement errors under different structure parameters: (**a**) the angle α between optical axis and baseline; (**b**) focal length f; (**c**) baseline distance B; (**d**) monitoring distance Z.

Ideally, the five points corresponding to the same abscissa should coincide, that is, the displacement values measured at the same position should be the same. However, it can be seen from Figure 5 that some groups of data showed a large deviation. This was due to other effects during the monitoring process, such as imperfect instrument structures and improper camera shutter operation. Therefore, in the subsequent analysis of the error distribution curve, we filtered out the images with larger relative error data. Finally, Equation (12) was used to calculate the root mean square error (RMSE) of each group of data to evaluate the deviation between the observed value and the true value, and the error distribution curve was obtained as shown in Figure 6.

(12) $$E=\sqrt{\frac{1}{n}\sum_{i=1}^{n}{\left(d_{i}-d_{t}\right)}^{2}},$$

where $d_{t}$ is the true displacement value; n is the amount of valid data measured for each group.

Figure 6a expounds the variation of error with different angles between the optical axis and baseline. Between 35° and 80°, the general variation trend of error increased with the increase of angles. At 45 °, the error reached a minimum. It could be considered that the proposed BVMS was more reliable for monitoring when the angle is less than 50°. It can be apparently seen from Figure 6b that the focal length was inversely proportional to the monitoring error. As the focal length increased, the field of view narrowed, so there is relatively little interference information in the image. Therefore, the camera lens with large focal length as far as possible should be arranged in practical applications. The third group focused on the effect of the baseline distance on the error. In BVMS, the baseline distance is an important parameter and is often considered in conjunction with the monitoring distance. As shown in Figure 6c, the overall trend of error decreased first and then increased. When the ratio of the baseline distance to the monitoring distance was between 0.9 and 1.2, the error got a smaller value and the result seemed to be acceptable. Figure 6d illustrates the relationship between the monitoring distance and the error. As the monitoring distance increased, we can clearly see that the error also increased. Although the measured displacement error may break through 8 mm when the monitoring distance exceeds 50 m, it is believed that with the development of vision sensors, the monitoring distance of the BVMS will be more and more optimized, and remote, high-precision monitoring will be realized.

### 3.3. Sensitivity Analysis and Optimization Design

The influence degree of different system structure parameters on binocular measurement results was different. If the sensitivity of each parameter can be determined, the high-sensitivity parameter can be quickly and effectively suppressed and the monitoring accuracy can be improved.

For sensitivity analysis, the parameter combination set should be established first, as listed in Table 1. The value range of each parameter was determined according to the previous analysis and the tests. In addition, since different structure parameters have different units of physical quantities, it was difficult to rank the sensitivity degree, so the sensitivity factors of each parameter needed to be normalized dimensionless [43], as shown in Equation (13).

(13) $$S\left(a_{k}\right)=\max\left\{\left[\frac{f{\left(a_{k}\right)}_{\max}-f\left(a_{k}^{*}\right)}{f\left(a_{k}^{*}\right)}\right],\left[\frac{f\left(a_{k}^{*}\right)-f{\left(a_{k}\right)}_{\min}}{f\left(a_{k}^{*}\right)}\right]\right\},$$

where $S\left(a_{k}\right)$ is the normalized sensitivity factor of the evaluation parameter $a_{k}$; $f\left(a_{k}^{*}\right)$ corresponds to the reference value of system accuracy; $f{\left(a_{k}\right)}_{\max}$ and $f{\left(a_{k}\right)}_{\min}$ are respectively the maximum and minimum values of the corresponding accuracy within the value range of system parameter $a_{k}$.

**Figure 7:**
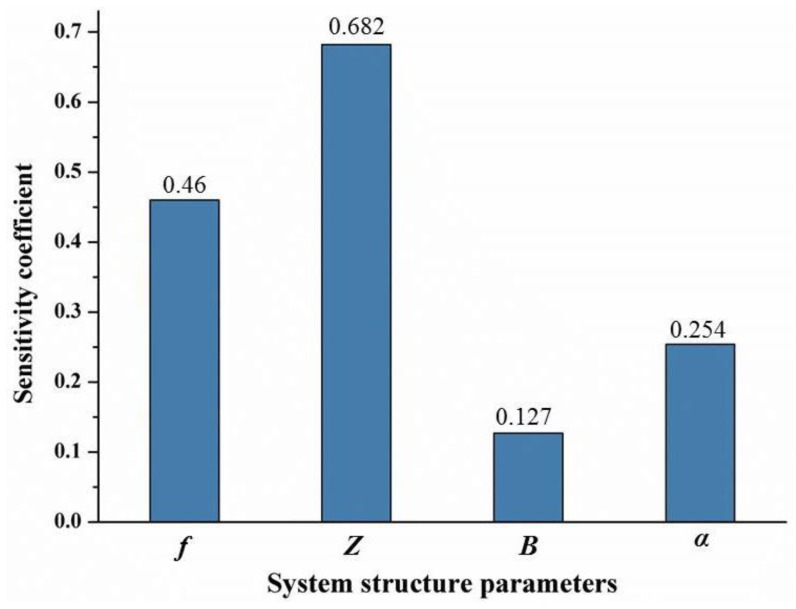
The sensitivity coefficient of the system structure parameters.

According to the error synthesis theory [42], the value of function $f\left(a_{k}\right)$ is equal to the sum of errors caused by each parameter. The error range caused by baseline distance and the angle between optical axis and baseline could be deduced from Equations (7)–(10). Focal length and monitoring distance affect the system accuracy by affecting the actual physical size represented by the pixel points, and the generated error $\Delta_{fZ}$ could be calculated by Equation (14) [44]. Finally, the sensitivity coefficient of each parameter could be obtained, as shown in Figure 7.

(14) $$\Delta_{fZ}=\frac{ZC}{f},$$

where C represents the size of pixel points, which was set as 6.25 μm in our experiment.

Figure 7 shows that the sensitivity coefficients of two parameters, the monitoring distance Z and the camera focal length f, were significantly higher than the other parameters, which means these two parameters had a larger influence degree on the total monitoring error than the other parameters. Therefore, in the application of BVMS, the two parameters should be efficiently configured according to the specific situation to reduce the monitoring error.

In order to facilitate the application of engineering monitoring, we designed the corresponding binocular system structure parameters combinations according to the former analysis, as listed in Table 2. In engineering applications, appropriate cameras and structure parameters combination can be selected according to specific precision requirements, monitoring scene and project budget.

## 4. Accuracy Analysis of Environment Parameters

In the vision-based slope monitoring, the main work is to process the acquired slope images. Landslides occur frequently in the rain, fog and other weather conditions, resulting in the images containing complex environment parameters. If these interferences can be filtered rapidly and effectively, it would be helpful for extracting objects and image matching and positioning, and then improve the accuracy and efficiency of slope deformation monitoring.

### 4.1. The Influence of Each Parameters

**Figure 8:**
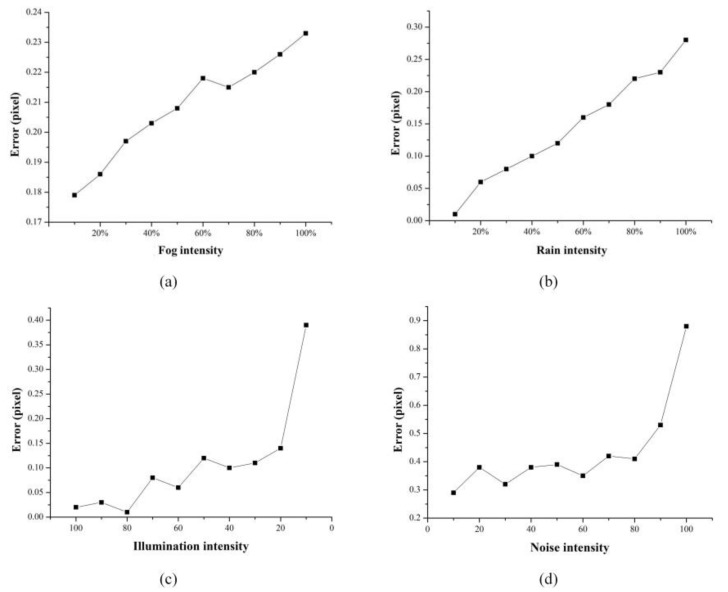
The influence of environment parameters on image center positioning results: (**a**) fog; (**b**) rain; (**c**) low illumination; (**d**) noise.

Simulation tests were designed in this section to study the influence of four common environmental factors on the image center positioning results. The interference of each environment parameter was simulated by adding filters, adjusting color levels, modifying layer modes and other basic operations in Photoshop. The center point positioning result of the disturbed image was compared with that of the original image, and the error distribution curve was obtained, as shown in Figure 8. The size of all images was 1095 × 956 pixels, and the central coordinate of the original image was (466.977, 565.527).

For the simulation of fog, we changed the intensity of fog by adjusting the filling amount of fog in the image. As can be seen from Figure 8a, the larger the fog, the greater the image positioning error. In foggy environments, the fog and dust permeated in the air will blur vision, and the image obtained under foggy conditions is significantly degraded, and the features such as the contrast and color of the target are attenuated, thus reducing the accuracy of target positioning.

Similarly, the simulation of rain with different intensity was realized by changing the filling amount of rain. Figure 8b presents that the error is positively correlated with the amount of rain. The impact of rain on the imaging quality was mainly concentrated in three aspects. One was that the raindrops blocked the target to a certain extent, making it difficult to accurately locate the target in the image. The second was that the existence of rain lines caused errors in the detection of target edges. Third, the image became blurred, which reduced the robustness and adaptability of the vision monitoring system, and was not conducive to subsequent target positioning and calculation.

The simulation of low illumination was relatively simple; we just changed the brightness value of the image directly. Figure 8c shows that although the error distribution curve fluctuates, the general trend is that the error increases with the decrease of brightness. In the case of poor lighting conditions, such as dusk and dawn, the imaging quality was severely deteriorated, mainly due to the large dark area of the image, blurred content and loss of details. Therefore, it is difficult to meet the needs of monitoring systems.

Noise simulation was achieved by adding filters, and the intensity of the added noise could be set. Through a large number of experiments, we found that the effect of noise on image positioning was the greatest. Figure 8d illustrates the relationship between noise intensity and positioning error: the higher the noise intensity, the more unnecessary or redundant interference information in the image data. In the field of monitoring, the error caused by noise will be amplified in subsequent processing, and with the transmission of errors, the results will show a non-negligible error.

### 4.2. Optimization Design

**Figure 9:**
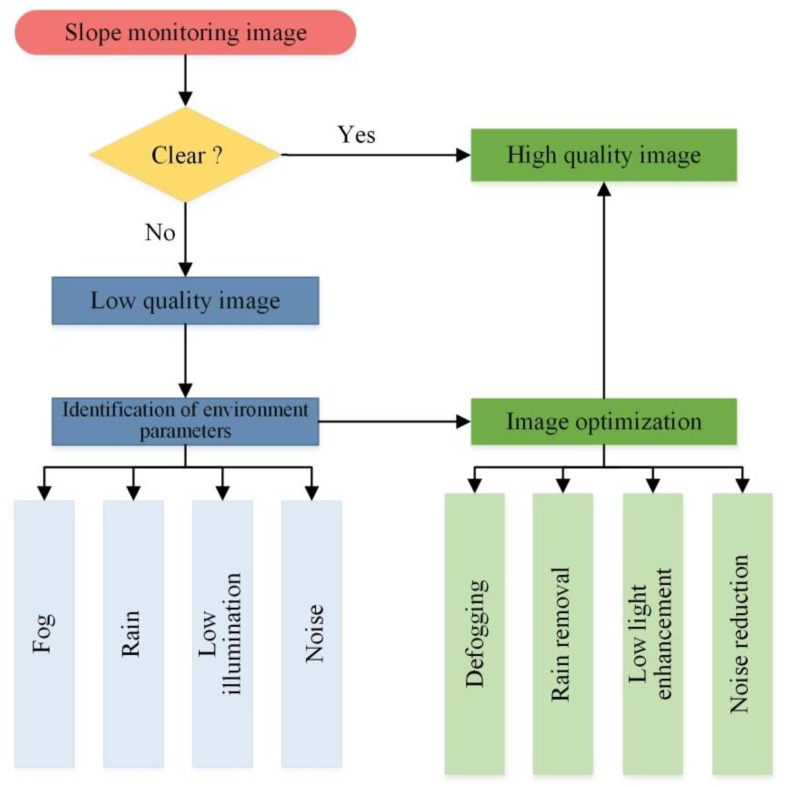
The process of slope monitoring image optimization.

From the previous analysis, it can be seen that the interference of environment parameters will cause deviations in image positioning. If the image cannot accurately reflect the actual situation of the slope measurement points, it is difficult to quantify the deformation. Therefore, this paper designed the corresponding image processing technology for the monitoring images under the above four types of environmental interferences to achieve image optimization (see Figure 9).

(1) Fog

Fog degrades the collected images, affects the positioning accuracy and even loses the target points. Therefore, the dark channel prior technology [45] was adopted to remove haze from images. On this basis, the guided filtering [36] was used to refine the transmittance, so as to achieve image defogging.

(2) Rain

Rainfall decreases the image contrast and blurs the target. On the account of deep learning [46], a neural network model [47] was employed to remove the rain streaks.

(3) Low illumination

Insufficient light will cause the captured image to have a large area of dark areas, which is difficult to meet the requirements of subsequent processing. In this paper, image main structures extraction technology [48] and low-illumination image degradation model [49] were used to enhance low light images.

(4) Noise

Interference of various factors induces noise in the image process of capture and transmission. In succession, the noise error will be continuously amplified in the subsequent processing. The Block-Matching and 3D filtering (BM3D) algorithm [50] was adopted to reduce image noise. Based on the self-similarity of images, the algorithm evaluated the similarity between blocks of images at different positions, so as to estimate and restore the original gray level of image pixels.

### 4.3. Performance Verification

This section describes two tests to verify the image optimization method proposed in this study.

(1) Interference of single environment parameter

**Figure 10:**
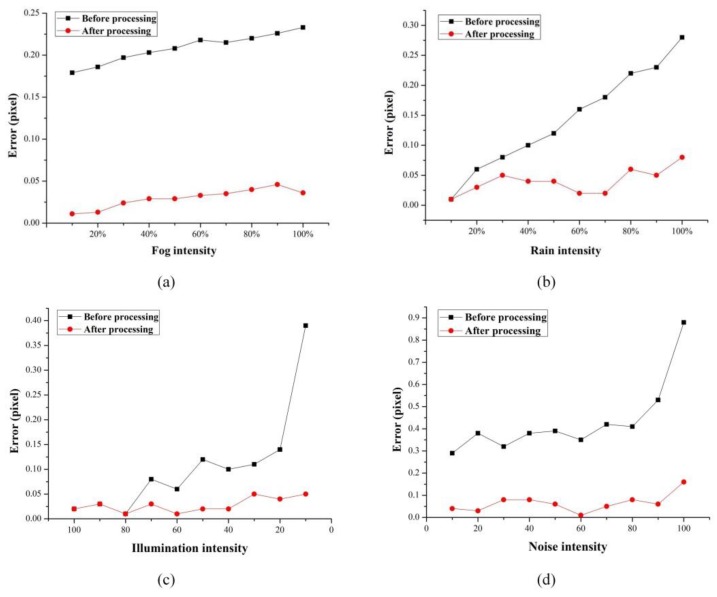
Target center positioning errors under single environment parameter interference before and after processing: (**a**) fog; (**b**) rain; (**c**) low illumination; (**d**) noise.

In the previous study of the influence of each environment parameters on the center positioning results, images were obtained under each environment interference. The corresponding optimization methods were used for processing, and the optimized image center positioning results were compared with the original image to obtain a processed error distribution curve, as shown by a red curve in Figure 10. The black curve is the error distribution curve before processing.

It can be clearly seen from the Figure 10 that the interference of the four environment parameters leads to the positioning deviation. However, for the effects of fog, rain and low illumination, the maximum error of the positioning results before processing did not exceed 0.4 pixels, indicating that the proposed BVMS has good robustness. Although the error was relatively large when the noise intensity was high, the positioning accuracy was significantly improved after optimization. On the whole, the maximum error of the optimized positioning result was 0.146 pixels, and only one group of pixel errors exceeded 0.1. It shows that the designed image optimization methods can effectively improve the influence of environmental parameters on the monitoring images.

(2) Interference of combined environment parameters

**Figure 11:**
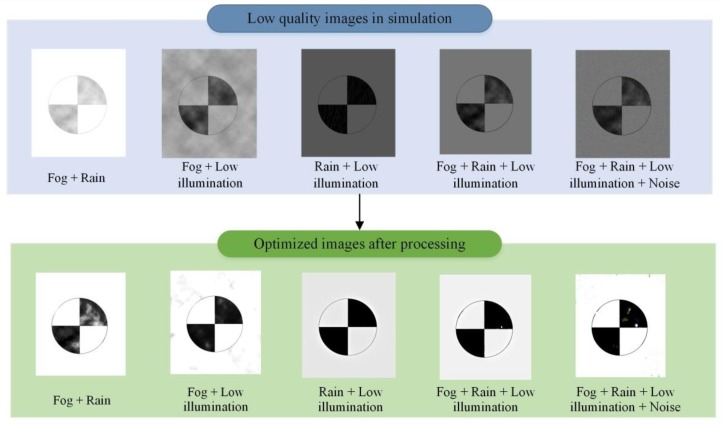
The simulated images before and after processing under the combined interference of multi-environment parameters.

Actually, the images were often affected by more than one types of environment interference, so it is of great significance to study the interference of multi-environment parameters. The following five sets of simulation tests are designed for slope monitoring. Figure 11 shows images before and after processing. It is worth noting that the interference intensity of identical environment parameters was the same during the testing of different combinations. That was achieved by keeping the parameters involved in the simulation consistent in Photoshop. The center point positioning was also performed on the images before and after optimization. The error results are listed in Table 3.

As shown in Figure 11, under the interference of combined environment parameters, the image quality seriously degraded, and the target area was easy to blur. This is likely to result in positioning failure. Judging from the location results before processing (see Table 3), in general, the more environment parameter interferences contained in the image, the greater the positioning error. When optimizing, low illumination handling was the most challenging. Different optimization parameters had to be set for processing, and the best-performing images were selected for subsequent analysis. In addition, by comparing a large number of test data, we found that different processing orders lead to different positioning results. Optimizing the factors that are easy to identify first, and then processing the factors that are difficult to discern, can make the unrecognized images get better positioning. In short, the optimized positioning error was no more than 0.11 pixels, which indicates that the optimization methods proposed in this paper can be well used for processing slope-monitoring images.

## 5. Field Tests

After optimizing the binocular measurement system and realizing the deformation monitoring of the target, we conducted further field tests. The deformation of the slope surface was simulated in the field and the applicability and accuracy of the method proposed in this paper was verified by the comparative analysis of the monitoring data.

**Figure 12:**
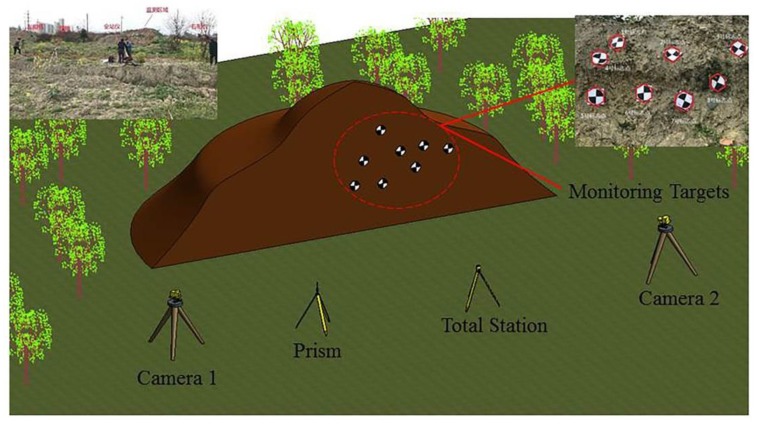
General arrangement of field tests.

In total, eight target points were set randomly on the slope surface, as shown in Figure 12. In order to verify the efficiency of the proposed BVMS, the surface deformation of the slope was simulated by artificially moving these points and compared with the measurement results of the total station. The combination of structure parameters is listed in Table 4. The influence of environment parameters was optimized before calculation.

Comparing the measurement results of the total station and BVMS, the root mean square errors of the eight targets in the X, Y, and Z directions and the total deformation were obtained, as shown in Table 5.

We can conclude from Table 5 that the BVMS and method proposed in this paper can be well applied to structural deformation monitoring. The final measured error is within the range of theoretical error, which also verifies the practicability of the proposed structure parameters combination recommendation table (see Table 2). Although the current results show that the monitoring distance is best controlled within 60m, it can already be used for monitoring of small slopes and small foundation pit engineering. In addition, with the development of binocular vision technology, its application range and monitoring distance will be more improved.

## 6. Conclusions

In this paper, the factors influencing the accuracy of the BVMS in slope monitoring are analyzed systematically. The conclusions can be drawn as follows:

(1) By combining the principle of binocular vision technology and the error analysis theory, the functional relationship between structure parameters and monitoring results was studied. Further laboratory tests were performed, and the error distribution curves under the influence of the angle between optical axis and baseline, focal length, baseline distance, and monitoring distance were obtained, respectively. Furthermore, the recommendation of structure parameters combination using the BVMS was also proposed.
(2) Through the environmental interference simulation tests, the error distribution curves under the influence of fog, rain, low illumination and noise were obtained. Therefore, image optimization methods are designed to deal with the interference of these four environment parameters. It relies on modifying the pixel distribution of the image to improve the contrast of the target, so as to further improve the environmental adaptability of the proposed system.
(3) Finally, the slope movement is simulated on site, and the deformation is monitored employing the proposed method. The results not only verify the validity of the structure parameters combination recommendation table, but also reflect the application potentials of the BVMS for cost-effective slope health monitoring. However, its applied range and monitoring distance still need to be improved.

## Figures and Tables

**Table 1:** The structure parameters combination set for sensitivity analysis.

Parameters	Focal Length f (mm)	Monitoring Distance Z (m)	Baseline Distance B (m)	The Angle α Between Optical Axis and Baseline (°)
Reference value	100	40	40	45
Value range	50–300	20–100	32–48	30–50
Step length	50	20	4	5

**Table 2:** Recommendation of structure parameters combination using the binocular vision measurement system.

Error (mm)	Focal Length f (mm)	Monitoring Distance Z (m)	Baseline Distance B (m)	Angle α Between Optical Axis and Baseline (°)
4.8–7.7	50	20	16–24	30–50
3.55–6.45	100	20	16–24	30–50
3.13–6.03	150	20	16–24	30–50
2.92–5.82	200	20	16–24	30–50
2.72–5.62	300	20	16–24	30–50
7.29–10.19	50	40	32–48	30–50
4.8–7.7	100	40	32–48	30–50
3.96–6.86	150	40	32–48	30–50
3.55–6.45	200	40	32–48	30–50
3.13–6.03	300	40	32–48	30–50
9.79–12.69	50	60	48–72	30–50
6.05–8.95	100	60	48–72	30–50
4.8–7.7	150	60	48–72	30–50
4.17–7.07	200	60	48–72	30–50
3.55–6.45	300	60	48–72	30–50
12.29–15.19	50	80	64–96	30–50
7.29–10.19	100	80	64–96	30–50
5.63–8.53	150	80	64–96	30–50
4.8–7.7	200	80	64–96	30–50
3.96–6.86	300	80	64–96	30–50
14.79–17.69	50	100	80–120	30–50
8.54–11.44	100	100	80–120	30–50
6.46–9.36	150	100	80–120	30–50
5.42–8.32	200	100	80–120	30–50
4.38–7.28	300	100	80–120	30–50

**Table 3:** The positioning results of target center before and after image processing.

Number	Types of Environment Parameter	Error before Processing (pixels)		Error after Processing (pixels)	
		x	y	x	y
1	Fog + Rain	0.17	0.075	0.049	0.066
2	Fog + Low Illumination	0.092	0.027	0.012	0.029
3	Rain + Low Illumination	0.011	0.056	0.08	0.025
4	Fog + Rain + Low Illumination	0.183	0.085	0.038	0.006
5	Fog + Rain + Low Illumination + Noise	0.515	0.721	0.11	0.042

**Table 4:** The designed combination of structure parameters.

Focal Length f (mm)	The Angle α Between Optical Axis and Baseline (°)	Monitoring Distance Z (m)	Baseline Distance B (m)	Ratio k of Baseline Distance to Monitoring Distance
100	45	20	24	1.2
		40	48	
		60	72	
		80	96	

**Table 5:** The displacement error results measured by binocular vision measurement system.

Monitoring Distance Z (m)	X Direction Error (mm)	Y Direction Error (mm)	Z Direction Error (mm)	Total Error (mm)
20	2.04	1.82	3.21	4.15
40	4.20	3.76	3.18	6.47
60	4.81	4.82	4.77	8.31
80	7.13	5.97	7.04	11.67
